# Retrospective analysis of genetic etiology and obstetric outcome of fetal cystic hygroma: A single-center study

**DOI:** 10.1097/MD.0000000000031689

**Published:** 2022-11-25

**Authors:** Meiying Cai, Nan Guo, Na Lin, Hailong Huang, Liangpu Xu

**Affiliations:** a Medical Genetic Diagnosis and Therapy Center, Fujian Maternity and Child Health Hospital, College of Clinical Medicine for Obstetrics & Gynecology and Pediatrics, Fujian Medical University, Fujian Key Laboratory for Prenatal Diagnosis and Birth Defect, Fuzhou, China.

**Keywords:** cystic hygroma, SNP-array, copy number variations, obstetric outcome

## Abstract

Cystic hygroma (CH) is a relatively common observation in prenatal ultrasounds; however, there are few studies about copy number variations (CNVs) of fetuses with CH.

**Methods:**

We performed a retrospective analysis on 40 pregnant patients (out of 8000 pregnant patients) whose fetuses had CH from November 2016 to June 2021. Villus, amniotic fluid, or umbilical cord blood samples were collected, based on the corresponding gestational age, for karyotype analysis and single-nucleotide polymorphism array (SNP-array).

**Results:**

Among the 40 fetuses with CH, 16 (40.0%, 16/40) exhibited isolated CH and 24 (60.0%, 24/40) exhibited CH combined with other ultrasound abnormalities. The most common CH-comorbid ultrasound abnormalities observed in this study were congenital heart disease (25.0%, 6/24), thickened nuchal translucency (20.8%, 5/24), and fetal edema (12.5%, 3/24). Karyotype and SNP-array analysis resulted in an overall detection rate of 30.0% (12/40). Karyotype analysis led to the detection of eight cases of pathogenic CNVs, among which 45, X was the most common. In addition to the above pathogenic CNV, four additional cases were detected by SNP-array. There was no significant difference in the observed pathogenic CNVs between isolated CH and CH combined with other ultrasound (31.3% vs 29.2%, *P* > .99). Karyotype analysis and SNP-array results influence whether parents terminate the pregnancy. When genetic abnormalities are detected in the fetus, the parents often choose to terminate the pregnancy.

**Conclusions:**

Our study emphasizes that genomic examination should be performed on fetuses with CH to confirm the etiology as soon as possible. During genetic counseling, all fetal characteristics should be carefully and comprehensively evaluated.

## 1. Introduction

Cystic hygroma (CH) is a congenital abnormality of the fetal lymphatic system that has an approximate incidence of 1 in 6000.^[1,2]^ This lymphatic reflux disorder results in extreme cystic dilation of lymphatic vessels in the neck and limbs.^[3,4]^ The development of the fetal lymphatic system begins at 10 weeks of gestation and is completed by 14 weeks of gestation. Systemic lymphatic fluid reflux occurs in the neck of the thoracic and right lymphatic duct and then reverts to the venous system. Regardless of the cause of fetal venous pressure, lymphatic reflux obstruction can cause fetal water sac changes in the neck and entire body. CH usually occurs in the head and neck but may affect the armpit, mediastinum, limbs, and other parts of the body.^[5,6]^

Continued improvements in ultrasound resolution have made detection of fetal abnormalities during pregnancy easier and have also improved the detection rate of CH. However, the prognosis of CH, which is affected by chromosomal abnormality and fetal malformation,^[7–11]^ remains poor.^[12–16]^ The identification of fetal CH increases the likelihood of termination of pregnancy by the patient. Many clinicians have recognized the importance of ultrasonic screening for genomic abnormalities in guiding healthcare for fetuses with CH during pregnancy. However, there are few studies related to copy number variations (CNVs) in fetuses with CH. Therefore, in this study, we retrospectively analyzed the pregnancy outcomes of 40 fetuses with CH and explored the relationship between CH, genomic abnormalities, and pregnancy outcomes. Our study provides evidence for improved and efficient clinical management of CH.

## 2. Materials and methods

### 2.1. Retrospective sampling

A total of 40 fetuses with CH (in over 8000 fetuses) were identified at the Fujian Maternal and Child Health Hospital, China from November 2016 to June 2021. Villus, amniotic fluid, or umbilical cord blood samples were collected according to the corresponding gestational age. Among the total samples (40), 24 were obtained via a puncture through the amniotic sac, ten via puncture through the umbilical cord, and six via puncture through the villi. The fetuses were divided into two groups based on the presentation of CH: fetuses with CH alone (16 cases) and fetuses exhibiting CH combined with other ultrasound abnormalities (24 cases). The mean age of the pregnant women was 26.4 years and the mean gestational age was 24.2 weeks. The testing and reporting procedures were approved by the ethics committee of Fujian Maternal and Child Health Hospital (no. 2014042).

### 2.2. Traditional karyotype analysis

The 40 isolated villi, amniotic fluid, and cord blood samples were cultured, harvested, dropped, and stained according to the G-banding karyotype analysis technique established by our center.^[17]^ Karyotypes were collected and analyzed using the automatic chromosome scanning platform. A total of 40 karyotypes were counted in each case, out of which five were analyzed. The number of karyotypes counted and analyzed was increased in the presence of an abnormality.

### 2.3. Single-nucleotide polymorphism array (SNP-array)

SNP-array was performed on the samples isolated from 40 fetuses. Digestion, amplification, purification, fragmentation, labeling, hybridization, washing, scanning, and data analysis of genomic DNA samples were performed by strictly following the standard procedures provided by Cytoscan 750K (Affymetrix, Santa Clara, CA, USA). The corresponding CHAS software and bioinformatic methods were used to analyze the SNP-array results. CNVs were determined according to the scatter plot distribution of DNA fragment copy numbers. The detected CNVs were compared and analyzed based on the public database. According to the guidelines of the American Society of Medical Genetics and Genomics,^[18]^ CNVs were categorized as follows: pathogenic, likely pathogenic, having variations of uncertain clinical significance, likely benign and benign.

### 2.4. Statistical analysis

Statistical analysis was performed using SPSS 21.0 software and Fisher exact probability test was used for comparison of the rate of detection of pathogenic CNVs between the two groups. The differences with *P* < .05 were considered statistically significant.

### 2.5. Follow up

Outcome of pregnancy and postpartum growth and development were determined by follow-up telephonic appointments.

## 3. Results

### 3.1. Fetal profile

Among the 40 fetuses with CH, 16 (40.0%, 16/40) had isolated CH and 24 (60.0%, 24/40) had CH combined with other ultrasound abnormalities. In fetuses with CH combined with other ultrasound abnormalities, the most common were congenital heart disease (25.0%, 6/24), thickened nuchal translucency (20.8%, 5/24), and fetal edema (12.5%, 3/24) (Table 1).

**Table 1:** Phenotypic characteristics of fetuses with CH with other ultrasound abnormalities.

Classification	Number of fetuses
CH with congenital heart disease	6
CH with thickened nuchal translucency	5
CH with edema	3
CH with dissociation of the renal collecting system	2
CH with nasal bone hypoplasia	2
CH with polyhydramnios	1
CH with digestive malformation	1
CH with tricuspid regurgitation	1
CH with abnormal venous catheter wave	1
CH with multiple system malformation	2
Total	24

CH = cystic hygroma.

### 3.2. Results of karyotype analysis

Chromosomal karyotype analysis was performed successfully in all 40 fetuses with CH, and a total of eight cases (20.0%, 8/40) were identified as abnormal. Among them, five cases were of 45, X, two cases had trisomy of chromosome 21, and one case had abnormal chromosomal structure (Table 2).

**Table 2:** Results of karyotype analysis in fetuses with CH.

Case	Karyotype analysis	Ultrasonic phenotype	P classification	Obstetric outcome
1	45, X	CH	P	TP
2	45, X	CH	P	TP
3	45, X	CH, thickened nuchal translucency	P	TP
4	45, X	CH, congenital heart disease, edema	P	TP
5	45, X	CH, congenital heart disease, edema	P	TP
6	47, XY,+21	CH	P	TP
7	47, XY, +21	CH, congenital heart disease, nasal bone hypoplasia	P	TP
8	46,XY,add(4)(q35)	CH, abnormal venous catheter wave	P	TP

CH = cystic hygroma, P = pathogenic, TP = termination of pregnancy.

### 3.3. Results of SNP-array

SNP-array was performed on 40 fetuses with CH, and 12 cases (30.0%, 12/40) were identified as having pathogenic CNVs. In addition to the eight cases of pathogenic CNVs (which were consistent with karyotype analysis), SNP-array detected four additional cases of pathogenic CNVs. The additional pathogenic CNV cases included 1q21.1 microduplication, 15q11.2 microdeletion, 22q11.21 microdeletion, and 17p11.2 microdeletion (Table 3).

**Table 3:** Results of SNP-array in fetuses with CH.

Case	SNP-array results	Size (Mb)	Ultrasonic phenotype	P classification	Inheritance	Obstetric outcome
1	arr[hg19]1q21.1q21.2(145,958,361-147,830,830) × 3	1.8	CH	P	-	TP
2	arr[hg19] 15q11.2(22,770,421-23,082,237) × 1	0.3	CH	P	-	TP
3	arr[hg19] 22q11.21(18,631,364-21,800,471) × 1	3.1	CH, congenital heart disease	P	-	TP
4	arr[hg19] 17p11.2(16,727,490-20,433,723) × 1	3.7	CH, congenital heart disease, nasal bone hypoplasia, polyhydramnios	P	*de novo*	Postnatal death

CH = cystic hygroma, P = pathogenic, SNP = single-nucleotide polymorphism, TP = termination of pregnancy.

### 3.4. Comparison of pathogenic CNVs between CH and CH combined with other ultrasound abnormalities

In the isolated CH group, five cases of pathogenic CNVs were detected with a positive rate of 31.3%. In the CH with other ultrasound abnormalities group, seven cases of pathogenic CNVs were detected with a positive rate of 29.2%. There was no statistically significant difference in pathogenic CNV detection between the two groups (*P* > .99), as shown in Table 4.

**Table 4:** Difference in pathogenic CNV detection between the two groups.

Classification	Number of fetuses	Pathogenic CNVs
Isolated CH	16	5 (31.3%)
CH with other ultrasound abnormalities	24	7 (29.2%)
Total	40	12 (30.0%)

CH = cystic hygroma, CNV = copy number variation.

### 3.5. Obstetric outcome and follow-up

Among the 40 fetuses with CH, we were successful in following-up with 39 cases. Among them, 12 cases survived with a survival rate of 30.8% (12/39) and 24 cases were terminated with a termination rate of 61.5% (24/39). Of the remaining cases, one fetus was stillborn and two suffered postnatal death (the overall mortality rate of 7.7%, 3/39), as shown in Table 5.

**Table 5:** Obstetric outcomes of 40 CH fetuses.

SNP-array results	Ultrasonic phenotype of the fetus	Survival	TP	Obstetric stillborn	Outcome postnatal death	No follow-up	Total
Normal	Isolated CH	7	3	1	0	0	11
	CH with other ultrasound abnormalities	4	11	0	1	1	17
pCNV	Isolated CH	1	4	0	0	0	5
	CH with other ultrasound abnormalities	0	6	0	1	0	7
Total		12	24	1	2	1	40

CH = cystic hygroma, pCNV = pathogenic copy number variation, SNP = single-nucleotide polymorphism, TP = termination of pregnancy.

## 4. Discussion

Fetuses with CH can be detected more efficiently with the improved application of prenatal ultrasound technology. Timely interventional prenatal diagnosis can determine the possible etiology of fetuses with CH. In this study, 40 fetuses with CH were examined, and prenatal diagnosis was performed based on karyotype analysis and SNP array. Karyotype analysis revealed that the chromosome abnormality rate of the studied fetuses was 20.0% (8/40), which was lower than that reported in the literature.^[11]^ Of these, five cases exhibited Turner syndrome, two exhibited trisomy 21, and one exhibited chromosomal structural abnormalities. The primary abnormalities were consistent with those reported in the literature.^[7,19,20]^ Papp et al^[21]^ reported that the most common ultrasound phenotype in fetuses with Turner syndrome was CH. The presence of CH can be used as a soft indicator for screening chromosomal abnormalities in fetuses because they are at a significant risk of developing Turner syndrome and trisomy 21.

In addition to the eight cases of pathogenic CNVs (consistent with karyotype analysis), SNP-array detected four additional cases of pathogenic CNVs. One fetus with CH presented with a copy number loss of 22q11.21, which is approximately 3.1 Mb in size and contains 44 OMIM genes, including *TBX1*. This microdeletion resulted in 22q11.2 deletion syndrome.^[22]^ The clinical manifestations of this deletion include cardiac malformations, thymus hypoplasia, laryngopharyngeal atresia with cleft palate, and parathyroid dysfunction with hypocalcemia. Therefore, 22q11.21 microdeletion is considered pathogenic and clinically significant. The ultrasound phenotype of this fetus was cardiac malformation in addition to CH. Another fetus with CH had copy number loss of 17p11.2, which is approximately 3.7 Mb in size and contains 38 OMIM genes, including RAI1. This microdeletion results in Smith–Magenis syndrome.^[23]^ The main clinical features of this microdeletion are cognitive impairment, characteristic facial features, sleep disturbance, and increased risk of childhood obesity. Therefore, 17p11.2 microdeletion is considered pathogenic. In addition to CH, the ultrasound phenotype of this fetus also showed cardiac malformation, nasal bone dysplasia, and hydramnios. In another fetus, deletion of 1q21.1q21.2 was identified, which is approximately 1.8 Mb in size containing 23 OMIM genes. This region is the site of neurocognitive disorders, and patients with this microdeletion exhibit a variety of clinical phenotypes, including developmental delay, heart malformations, autism spectrum disorders, and schizophrenia.^[24]^ Therefore, the 1q21.1q21.2 microduplication is considered clinically significant and potentially pathogenic. The ultrasound phenotype of the fetus with 1q21.1q21.2 microduplication was isolated CH. One fetus with CH had the copy number loss of 15q11.2, which is approximately 312 Kb in size and contains 4 OMIM genes. The clinical phenotypes of patients with this variation vary widely, including developmental delay, feeding difficulties, and behavioral abnormalities.^[25]^ Therefore, 15q11.2 microdeletion is considered to have clinical significance and potentially pathogenic. The ultrasound phenotype of this fetus was isolated CH. SNP-array can accurately locate genomic abnormalities in fetuses, including copy number abnormalities, further clarifying the microdeletion and microduplication of small fragments near the breaking point. These analyses provide reliable information for clinicians to evaluate fetal development and prognoses, which is crucial for genetic counseling.

Heart malformation is the most common physiological abnormality that develops in fetuses with CH combined with other ultrasound abnormalities and is related to fetal heart failure.^[26]^ Some studies have reported that CH in the fetus is closely related to the thickened nuchal translucency. Prolonged accumulation of CH in the neck can also lead to edema.^[27,28]^ In this study, the most common additional ultrasound abnormalities observed were congenital heart disease, thickened nuchal translucency, and fetal edema, which was consistent with previous reports.^[14,29]^ The detection rate of pathogenic CNVs in fetuses with isolated CH was 31.3%, whereas that in fetuses with CH combined with other ultrasound abnormalities was 29.2%. There was no statistically significant difference between the two groups, which was inconsistent with previous reports.^[30,31]^ The observed difference might be related to sample size and population. The sample size and amount of data analyzed in this study is limited, and future studies with more extensive data and bigger sample size are required to further support our findings.

In terms of pregnancy outcome, the rate of full-term delivery of a fetus with CH fluctuates between 7.5% and 18.8%.^[13,15,32]^ In this study, the full-term delivery rate of fetuses with CH was 30.8%, which was higher than that reported in the literature. The prognosis of isolated CH with a normal genome is generally good under normal birth conditions. Some parents may consider termination in response to the identification of CH, which should be discussed by clinicians to avoid unnecessary and premature termination. Intrauterine therapy is a promising treatment for fetuses with CH.^[33]^ This study was limited by unavailability of treatment; therefore, further studies on managing fetal development after identifying CH should be conducted. Recently, a new genetic detection technology, next-generation sequencing, has been used to detect single gene mutations, which may provide more comprehensive prenatal genetic diagnoses for fetuses with CH and allow clinicians to better identify the disease and provide accurate prognoses.^[34,35]^

## 5. Conclusion

In conclusion, a genomic examination should be performed to identify the genetic etiology of CH during pregnancy. During genetic counseling, all fetal characteristics (such as genetic etiology, ultrasonic anomaly, and comorbidity with other ultrasound abnormalities) should be comprehensively evaluated, and ultrasound imaging should be regularly conducted to track changes in fetal phenotype. By establishing supportive and comprehensive prenatal clinical strategies, clinicians can better support parents whose fetuses exhibit potential abnormalities and aid in healthcare decision-making regarding pregnancy termination and postnatal development.

## Acknowledgments

We thank all patients for their participation.

## Author contributions

Liangpu Xu designed the study, Hailong Huang conducted the experiments, Na Lin and Nan Guo performed the data analysis, and Meiying Cai wrote the manuscript.

**Writing – original draft:** Meiying Cai.

**Data curation:** Nan Guo.

**Formal analysis:** Na Lin.

**Project administration:** Hailong Huang.

**Supervision:** Liangpu Xu.
